# Scalable Lead Acetate-Based Perovskite Thin Films Prepared via Controlled Nucleation and Growth under Near Ambient Conditions

**DOI:** 10.1021/acsomega.3c08912

**Published:** 2024-02-08

**Authors:** Saara Sirkiä, Muhammad Talha Masood, Mahboubeh Hadadian, Syeda Qudsia, Emil Rosqvist, Jan-Henrik Smått

**Affiliations:** † Laboratory of Molecular Science and Engineering, Åbo Akademi University, Henriksgatan 2, Åbo FI-20500, Finland; ‡ Department of Materials Engineering, School of Chemical & Materials Engineering, National University of Science & Technology (NUST), H 12 sector, Islamabad 44000, Pakistan; § Department of Mechanical and Materials Engineering, Faculty of Technology, University of Turku, Turku FI-20014, Finland

## Abstract

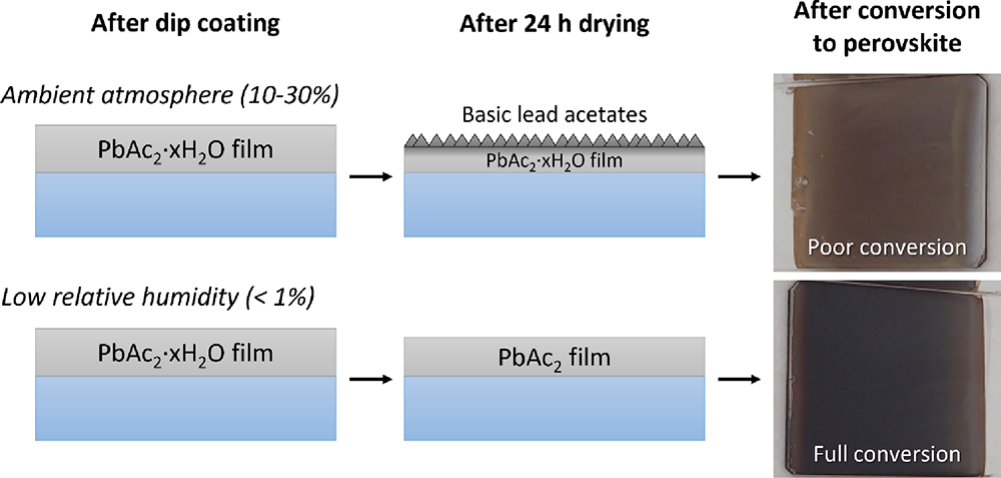

Lead acetate (PbAc_2_) is a promising precursor salt for large-scale production of perovskite solar cells, as its high solubility in polar solvents enables the use of scalable deposition methods such as inkjet printing and dip coating. In this study, uniform (40–230 nm) PbAc_2_ thin films were prepared via dip coating under near ambient lab conditions by tuning the PbAc_2_ precursor concentration. In a second step, these PbAc_2_ films were converted to methylammonium lead iodide (MAPI) perovskite by immersing them into methylammonium iodide (MAI) solutions. The nucleation and growth processes at play were controlled by altering key parameters, such as air humidity during the lead acetate deposition and MAI concentration when converting the PbAc_2_ film to MAPI. The research revealed that lead acetate is sensitive toward humidity and can undergo hydroxylation reactions affecting the reproducibility and quality of the produced solar cells. However, drying the PbAc_2_ films under low relative humidity (<1%) prior to conversion enables the production of high-quality MAPI films without the need of glovebox processing. Furthermore, SEM characterization revealed that the surface coverage of the MAPI film increased significantly with an increase of the MAI concentration at the conversion stage. The resulting morphology of the MAPI films can be explained by a standard nucleation and growth mechanism. Preliminary solar cells were produced using these MAPI films as the active layer. The best performing devices were obtained with a 140 nm thick lead acetate film converted to MAPI using a 12 mg/mL MAI solution, as these parameters resulted in a good surface coverage of the MAPI film. The results show that the methodology holds potential toward large-scale production of perovskite solar cells under near ambient conditions, which substantially simplifies the fabrication and lowers the production costs.

## 1 Introduction

The ever-increasing need for sustainable energy conversion applications has instigated a surge of the development of new solar cell technologies. One of technologies that harbors a vast potential is the perovskite solar cell (PSC). The power conversion efficiency of PSC devices has increased from the initial 3.8%^1^ to over 26%^2^ in just over a decade, comparable to the silicon solar cells in use today. PSCs carry high potential for use in various locations, not only limited to roof-top devices but having the possibility for integration into buildings,^3^ vehicles,^4−6^ or, as a semitransparent film, into windows.^7,8^ Perovskites comprise a wide variety of compounds with a general ABX_3_ structure,^9^ with A corresponding with a cation, for example, methylammonium (CH_3_NH_3_^+^), lead (Pb^2+^) representing B, and X for a halide anion, such as bromide (Br^–^), iodine (I^–^), or chloride (Cl^–^). The versatility of the octahedral structure makes it tunable in various properties and thus adaptable to different applications and device architectures. Methylammonium lead iodide (MAPI) is known to be one of the most used light-harvesting perovskite materials in photovoltaic applications.^10^ MAPI can be produced in numerous ways, and one of the most common laboratory scale deposition methods is spin coating. It is easy to perform, yet limited for the substrate size and, consequently, to the scalability of the sample size.^11^ Other deposition methods used for making perovskite layers include blade coating,^12^ inkjet printing, and screen printing.^13,14^ Dip coating holds a significant potential for the deposition of precursor solutions on a solid substrate as well as the conversion of precursor to MAPI, with the effortless and cost-effective nature of the method as well as its applicability for larger substrate sizes.^15,16^

The deposition of the precursor material and subsequent conversion into perovskite can be either performed in one single stage (one-step) or divided into two separate stages (two-step). In the one-step method, the precursor salts are dissolved individually in one common solvent, mixed, and deposited as one solution, for example, by spin coating^17^ or dip coating.^18^ To promote nucleation and growth of the perovskite crystals, different quenching methods can be used to quickly remove the solvent to create supersaturation of the perovskite precursors, including antisolvent quenching^19^ and gas quenching.^20^ Alternatively, the active layer can be deposited in two steps, where first the precursor layer is fabricated either by spin coating or dip coating and dried afterward. In the second stage, the precursor film is converted into MAPI by immersing the film into methylammonium iodide (MAI) or pipetting the converter solution on top while spin coating^21^ or by spin coating the converter solution on the precursor film.^22^ The conversion involves partial dissolution of the lead precursor and nucleation and growth of the perovskite layer. It has been suggested that there are two possible film formation mechanisms: at lower MAI concentrations, the crystal growth is more likely to occur at the solid–liquid interface, while with an increase in MAI concentration, a thin film of MAPI forms a blocking layer on the surface of the precursor film that inhibits conversion deeper in the precursor layer and leads easily to incomplete conversion.^23^ The two-step method is known to have several advantages over the one-step method such as resulting in more uniform surfaces with fewer pinholes and enabling the fabrication of films in a more humid environment. One of the drawbacks of using a two-step deposition method is the possibility of an incomplete reaction.^24^

The solubility of the lead precursors also limits the choice of lead salts that can be used in the dip coating process.^25,26^ Lead iodide (PbI_2_) has been widely studied as a precursor for PSCs, and in many studies, the reaction of PbI_2_ with MAI to form MAPI has been thoroughly investigated.^27^ However, as PbI_2_ is only soluble in solvents such as dimethylformamide (DMF) and dimethyl sulfoxide (DMSO), which have high boiling points and a slow evaporation rate, it is not an optimal precursor for the dip coating process. The choice of precursor salt also affects the reaction kinetics in the formation of the perovskite, and PbAc_2_ has been reported to have the fastest reaction rate when compared to lead chloride (PbCl_2_) and PbI_2_.^28,29^

Recent results have shown that producing MAPI using a solvent with a high boiling point in the precursor solution leads to a slower evaporation process that is deemed inapplicable to coating large areas with MAPI.^30^ Thus, dissolving the lead precursor salt into a solvent with a low boiling point, such as methanol, promotes faster evaporation of the solvent from the film and improves the film quality. Replacing commonly used toxic solvents such as *N*,*N-*dimethylformamide (DMF) with methanol has an additional positive impact on reducing the environmental burden of the process.^31^ Acetic acid (HAc), as an additive in the lead acetate solution, promotes better solubility of the metal salt at a lower pH.^32^ It has also been indicated that the presence of acetates in the perovskite precursor solution can improve the performance of the solar cell by an increase of the short-circuit current,^33^ making the precursor solution an optimal choice for a two-step deposition using dip coating in both deposition stages. Dip coating and immersion are the deposition methods for fabricating both the precursor film and final MAPI layer in this study.

Understanding the nature of the initial nucleation and the following crystal growth stage is crucial when aiming to produce the optimal MAPI film, and the most efficient way to control the structure and quality of the finished MAPI film is to steer the nucleation and growth of the MAPI crystals with key process parameters.^34^ By applying the La Mer model, both nucleation and crystal growth have been proven to be overlapping processes and thus compete over solute consumption during film formation.^35^ Earlier research on the crystallization control of lead iodide-based perovskite has been carried out by replacing methylammonium iodide with formamidinium iodide (FA).^36^ Promotion of higher MAPI quality and thus better device performance has also been studied using additives like azobenzene,^37^ and DMSO.^38^ Different methods for controlling the nucleation and growth process of perovskite films can be divided into physical approaches, such as thermal annealing, and chemical approaches concerning the modification of chemical compositions.^39^

The conversion process of lead acetate thin films to MAPI was investigated in this study. By investigating the reaction parameters, the nucleation and growth mechanisms during MAPI formation were clarified. It was observed that the mechanism follows expected nucleation and growth behavior when the lead acetate films are relatively thin (<100 nm) and the substrate coverage can be controlled by the MAI concentration. The light absorption and stability of the finished MAPI films were enhanced by drying the PbAc_2_ precursor films at a low RH to minimize the amount of physisorbed water in the MAPI film. Drying the films at low RH stabilizes the precursor film to such extent that the following conversion process can be performed in ambient conditions.

## 2 Experimental Section

### 2.1 Materials

TCO22-15 (also known as TEC 15) FTO glass substrates were purchased from Greatcell Solar Materials Ltd. The other chemicals purchased from the same manufacturer include cobalt(III) tri[bis(trifluoromethane)sulfonimide] (also known as FK 209 Co (III), >98%) and methylammonium iodide (MAI >98%). Titanium(IV) chloride was bought from Sigma-Aldrich. Ethanol (>99.5%) was purchased from ALTA Oyj. Lead(II) acetate trihydrate (PbAc_2_·3H_2_O, 99.999%), bis(trifluoromethane)sulfonimide (Li-TFSI), tri-s(2-(1*H*-pyrazol-1-yl)-4-*tert*-butylpyridine) (4-TBP), anhydrous methanol (99.8%), acetic acid (HAc), tetrahydrofuran (THF, >99%), and isopropanol (i-PrOH, anhydrous) were purchased from Sigma-Aldrich. 2,2′,7,7′-Tetrakis[*N*,*N*-di(4-methoxyphenyl)amino]-9,9′-spirobifluorene (Spiro-OMeTAD) was purchased from Luminescence Technology Corp. and gold pellets for thermal evaporation (99.999% pure) were purchased from Kurt J. Lesker Company.

### 2.2 PbAc_2_ Layer Deposition via Dip Coating

PbAc_2_ solutions were prepared by dissolving PbAc_2_·3H_2_O into a mixture of 9.8 mL of methanol and 0.1 mL of concentrated acetic acid. The experiments were carried out using solutions with four different concentrations (i.e., 0.24, 0.44, 0.63, and 0.81 M), and the samples were named accordingly: PbAc_2_-0.24M, PbAc_2_-0.44M, PbAc_2_-0.63M, and PbAc_2_-0.81M. These samples comprised a PbAc_2_ layer coated on top of plasma-activated glass substrates. They were prepared via the dip coating method using a withdrawal speed of 85 mm/min under ambient lab conditions (RH: 20–40%) or dry conditions (RH <1%) where they were kept for 24 h inside the dip coater chamber before further processing.

### 2.3 Conversion into MAPI

To produce the MAI converter solutions, 90–120 mg of methylammonium iodide was dissolved in 10 mL of isopropanol. The MAI chemical is stored inside the glovebox, and all the MAI solutions in i-PrOH have been prepared inside the glovebox. However, the conversion of PbAc_2_ to MAPI took place under ambient conditions (RH: 20–40%). The PbAc_2_ films were converted into MAPI by immersing a sample into the MAI solution for 60 s. Immediately after MAI treatment, the sample was dipped into 2-propanol to remove excess MAI from the film surface. The samples were then dried by spin coating at 4000 rpm for 10 s. Finally, the MAPI films were annealed by heating at 100 °C for 15 min.

### 2.4 Device Fabrication

The lab-scale solar cells were prepared on top of 2 cm × 4 cm FTO glass substrates, which were chemically etched from the top end using Zn powder and 4 M HCl solution. The dimensions of the etched region were approximately 1.5 cm × 2 cm. The etched substrates were ultrasonicated following the protocol by Saliba et al.^40^ The compact TiO_2_ films were produced following the protocol described by Masood et al.^41^

The PbAc_2_ films were deposited on the compact TiO_2_ layers and converted to MAPI as described earlier in Sections 2.2 and 2.3. The Spiro-OMeTAD solution was produced as reported by Saliba et al.^40^ The solution was spin-coated on the MAPI layers at 4000 rpm by pipetting 60 μL of solution onto a spinning sample and letting it spin for ≈10 s to dry. Finally, an 80 nm gold film was deposited as a back metal contact via thermal evaporation under a high vacuum.

### 2.5 Characterization

The absorbance of MAPI films was determined with a Lambda 1050+ UV–vis–NIR instrument (PerkinElmer, Waltham, MA, USA). Top-view images of MAPI films were captured with a Zeiss GeminiSEM 450 (Oberkochen, Germany) scanning electron microscope (SEM) using a secondary electron (SE) detector, as well as energy-dispersive spectroscopy (EDS) for elemental mapping. A Bruker Nanoscope V MultiMode 8 (Santa Barbara, CA, USA) atomic force microscope (AFM) was used to study the topology and surface coverage of the layers. Image analysis was done with MountainSPIP analysis software (v.9.3.10281, DigitalSurf, France). Imaging was done at *T* = 22 ± 2 °C and RH% = 40 ± 3 with NSG01 cantilevers, with a *k* = 5.1 (1.45 to 15.1) and a typical tip radius of 6 nm reported by the manufacturer (NT-MDT, Moscow, Russia). The analyzed images were of 10 × 10 and 1 × 1 μm size with a digital resolution of 512 × 512 pixels. Descriptions of the used roughness parameters are found in the Supporting Information. The thickness, roughness, and density of the PbAc_2_ films, as well as the crystal structure of the PbAc_2_ and MAPI films, were studied by using X-ray reflectometry (XRR) and X-ray diffraction (XRD) with a Bruker D8 Discover instrument (Karlsruhe, Germany). The XRR measurements were made in the 2theta range of 0.3°–3° with a step size of 0.01°. The data were analyzed using the DIFFRACplus LEPTOS software (v. 7.03, Bruker). The data signal was amplified and smoothed using the Origin 2022 software. XRD was measured between 5° and 40° 2theta with a step size of 0.04°.

## 3 Results and Discussion

### 3.1 Deposition of Lead Acetate via Dip Coating

Solutions with four different PbAc_2_·3H_2_O concentrations in methanol/acetic acid (0.24, 0.44, 0.63, and 0.81 M) were used to produce dip-coated films of various thicknesses on top of microscope glass substrates. The dip coating process and subsequent storage of the films (∼24 h) were made under very low relative humidity (<1%) provided by dry air flow inside the dip coating chamber. XRR measurements were performed to estimate the thickness, roughness, and density of the produced lead acetate films (see Table 1 and Figure S1 in the Supporting Information). The film thickness increased from 47 nm for the lowest concentration to 193 nm for the highest concentration. The surface roughness and film density for all samples were 0.4–0.5 nm and 3.14–3.20 g/cm^3^, respectively. Thus, the densities of these samples were close to the reported values for the anhydrous form of lead acetate (3.25 g/cm^3^),^42^ suggesting that the dry dipping (and storage) conditions favor the formation of the anhydrous form over the trihydrate form. This indicates that it is possible to produce very uniform anhydrous PbAc_2_ films simply by tuning the precursor concentration in the dip coating solution.

**Table 1:** Film Thickness, Roughness, and Density Values Were Extracted from XRR Measurements

**sample**	**thickness (nm)**	**roughness (nm)**	**density**(g/cm^3^)
PbAc_2_-0.24M	47	0.50	3.19
PbAc_2_-0.44M	78	0.51	3.19
PbAc_2_-0.63M	135	0.46	3.14
PbAc_2_-0.81M	193	0.43	3.20

For comparison, we also dip-coated lead acetate films under ambient lab conditions (typical RH range: 20–40%). Directly after dip coating, the produced films had a slightly lower density (∼2.9 g/cm^3^) based on XRR results, which increased up to ∼4 g/cm^3^ after storage for 24 h under ambient conditions. The reaction with water moisture also decreased the film thickness by about 10–15% compared to samples stored under dry conditions.

**Figure 1:**
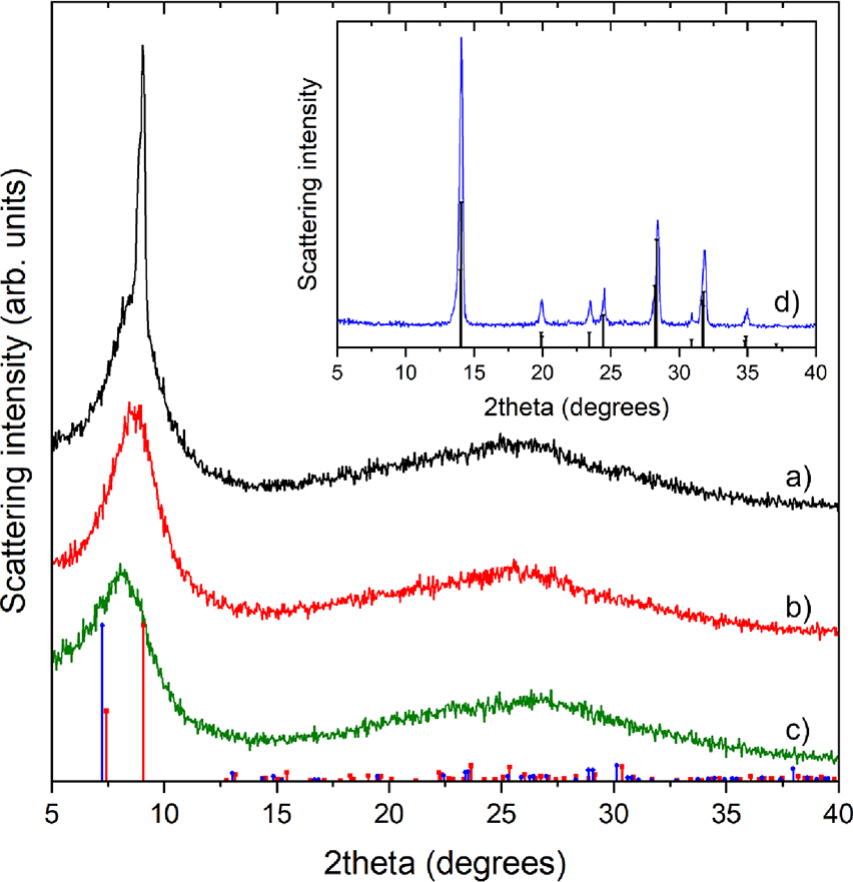
XRD patterns of PbAc_2_-0.63 M samples aged under (a) dry conditions for 24 h, (b) ambient conditions for 1 h, and (c) ambient conditions for 24 h. Reference patterns for anhydrous lead acetate (red, PDF card no. 00-018-1738) as well as 3PbAc_2_·PbO·H_2_O (blue, PDF card no. 00-018-1739) are included for comparison. Inset: (d) XRD pattern of the PbAc_2_-0.63 M sample converted to MAPI using an 11 g/mL MAI converter solution. The reference pattern of tetragonal MAPI is included for comparison (PDF card no. 00-068-0701).^45^

XRD measurements of PbAc_2_-0.63 M samples stored under dry conditions vs ambient humidity revealed more information about phase changes in the lead acetate film (see Figure 1). Figure 1a shows that the sample stored in the dip coating chamber (RH <1%) for 24 h has a sharp peak at 9.0° 2θ, which likely indicates larger crystallites of anhydrous lead acetate (PDF card no. 00-018-1738).^43^ Furthermore, a broad diffraction peak centered at around 8.7° 2θ is also present in the diffractogram, indicative of a more amorphous phase. For samples stored under ambient conditions (Figure 1b,c), only the broad diffraction peak can be observed. Moreover, the maximum of the peak shifts from 8.7° to 8.1° 2θ when the aging time of the film was extended from 1 to 24 h under ambient lab conditions. This could possibly indicate that the lead acetate reacted with water moisture to form amorphous basic lead acetates (e.g., lead acetate oxide hydrate). The data partially match the reference pattern of 3PbAc_2_·PbO·H_2_O (PDF card no. 00-018-1739),^43^ which is expected to have a sharp reflection at ∼7.2° 2θ. However, due to the noncrystalline nature of the sample and thus the broad peak, this is difficult to say for certain. It is interesting to note that the trihydrate form of lead acetate (PbAc_2_·3H_2_O) is not formed when drying the samples under ambient conditions, which has been observed when lead acetate is processed in a glovebox environment.^44^

AFM was used to investigate how humidity affected the topography of lead acetate films (see the Supporting Information, Figure S2 and Table S1). The surface of the sample stored under dry conditions (RH <1%) was very smooth, as seen from the low root-mean-square roughness value (*S*_q_) of approximately 0.32 nm and a developed surface area ratio (*S*_dr_) of approximately 0.01% (10 μm × 10 μm images). When stored under ambient conditions for 24 h (in this case, RH: ∼30%), the RMS height variations are significantly higher (*S*_q_ approximately 1.9 nm and *S*_dr_ approximately 0.17%). An extensive table of roughness parameters is found in the Supporting Information. From the topography images, the sample stored under dry conditions had a very narrow distribution of heights (approximately 2 nm in a 1 μm × 1 μm image), in line with the low *S*_q_. The sample stored in ambient condition had a less narrow distribution of heights, being approximately 5 nm for the core surface. The formation of island-like structures in the bottom 1–2 nm in the core surface could also be observed (Figure S2). These observations corroborate that lead acetate oxide hydrate has formed on top of the film. This alteration will have a profound effect on the subsequent conversion to MAPI, as will be discussed in the next section.

### 3.2 Conversion of PbAc_2_ into MAPI

**Figure 2:**
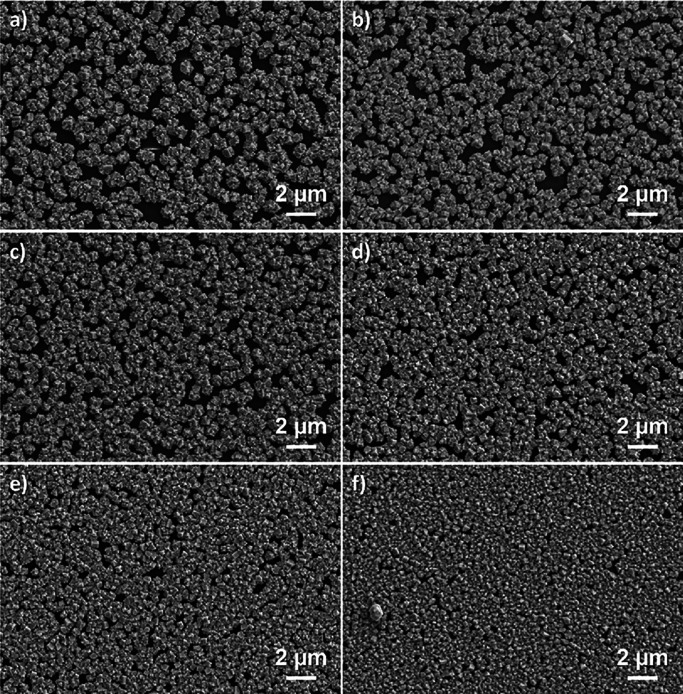
SEM images of PbAc_2_-0.63 M samples converted to MAPI in (a) 9.0 mg/mL, (b) 9.5 mg/mL, (c) 10.0 mg/mL, (d) 10.5 mg/mL, (e) 11.0 mg/mL, and (f) 12.0 mg/mL MAI solutions.

Lead acetate films (PbAc_2_-0.63M) aged under dry conditions were further converted into MAPI by immersion in solutions of MAI in i-PrOH at different concentrations. The conversion was done in ambient air (RH: 20–30%). A rapid color change from transparent to dark brown was observed, which indicates that the conversion process was very fast and occurred within a few seconds after immersion. SEM images show a gradual change in the MAPI structure and surface coverage upon increasing the MAI concentration from 9 to 12 mg/mL (see Figure 2 and Table S2). The surface coverage of MAPI on top of the glass substrates increased from 72 to 92%, while the grain size decreased from about 850 to 400 nm. Higher magnifications of the MAPI films (see Figure S3 in the Supporting Information) reveal that the samples consist of larger cuboid crystal grains surrounded by smaller particles (also cuboid in shape). This suggests that there are secondary nucleation processes on top of the original crystal grains, and thus, an overall rounded grain shape is observed. Similar observations for PbI_2_ (as the lead precursor) have been reported in earlier studies, which were directly correlated with the nucleation and growth process of the MAPI crystals/particles.^28,39^ The nucleation and growth mechanisms will be discussed in more detail in Section 3.3.

**Figure 3:**
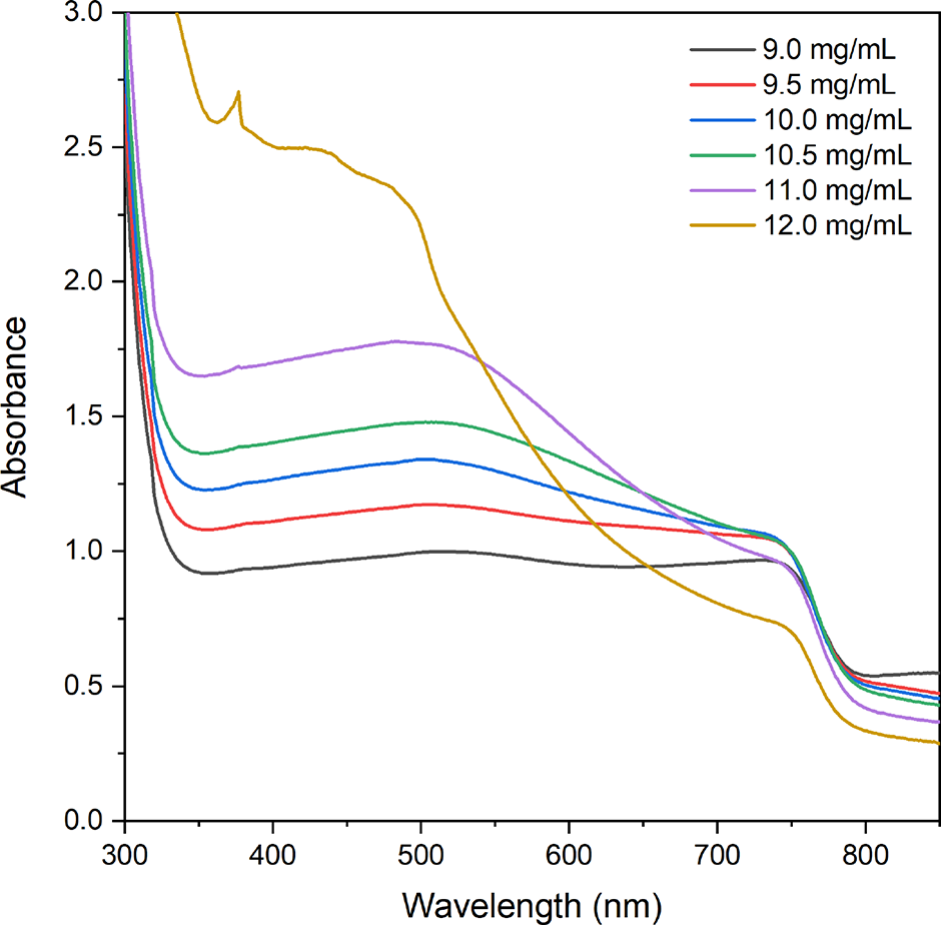
UV–vis absorption spectra for MAPI films on glass after the conversion of PbAc_2_-0.63 M samples in MAI solutions with various concentrations.

UV–vis spectroscopy measurements were performed to estimate the degree of conversion of lead acetate to MAPI upon increasing the MAI solution concentration. The UV–vis spectra in Figure 3 reveal that the absorbance at ∼750 nm is about 1 absorbance unit when using 9 mg/mL concentration of MAI solution. The absorbance at this wavelength intensified slightly upon increasing the MAI solution concentration to 9.5 mg/mL and remained at the same level until a significant decrease in absorbance at 750 nm was observed for the highest MAI concentration (12 mg/mL). The onset of absorbance at 770 nm is associated with the bandgap energy of MAPI present in the films, and the results indicate that less MAPI is formed for the highest MAI concentrations. Moreover, a continuous increase in the broad absorbance between 500 and 650 nm was observed when the MAI concentration was increased from 9 to 12 mg/mL. This increase in absorption is related to structural changes in the MAPI film (e.g., surface coverage, film thickness, and particle size).^46,47^ Furthermore, a steeper slope at 500 nm can be seen for the 12.0 mg/mL sample, indicating some degradation to PbI_2_.^48^

XRD measurements revealed that all unannealed MAPI samples have the same crystal structure (see example of the PbAc_2_-0.63 M sample converted to MAPI using an 11 g/mL MAI converter solution in Figure 1d). The peaks correspond to tetragonal MAPI, which should be formed at room temperature.^45^ After annealing at 100 °C for 15 min (under ambient conditions), all samples showed signs of the formation of lead iodide. This suggests that humidity in the air causes some degradation of the topmost part of the MAPI layer.

### 3.3 Formation Mechanism

**Figure 4:**
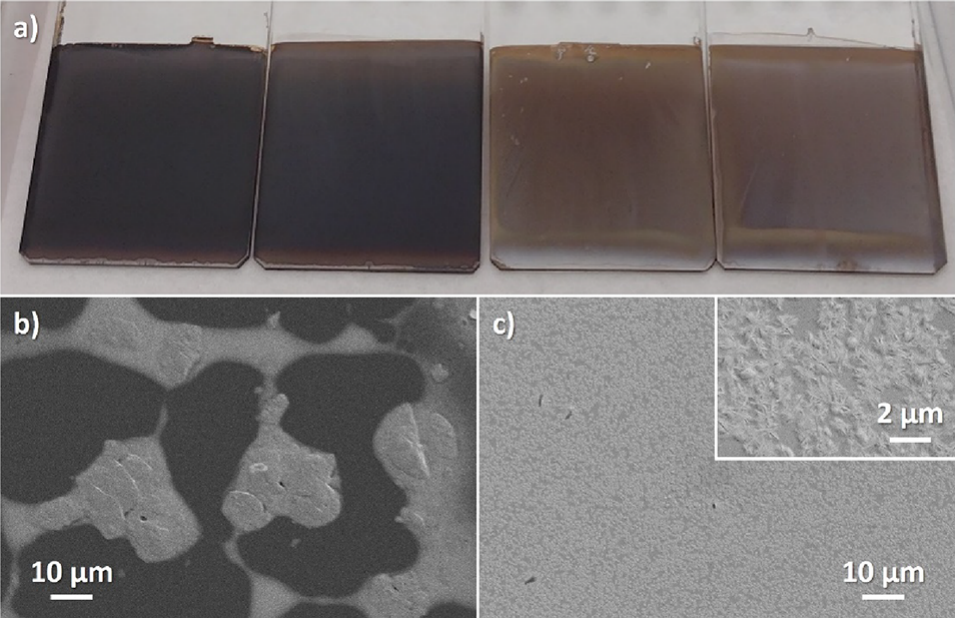
(a) MAPI films produced from lead acetate films stored in dry air (left) and under ambient laboratory conditions (right). SEM images of lead acetate films after storing/aging under a (b) dry or (c) ambient atmosphere for 24 h and then immersing them in i-PrOH.

To clarify the reaction mechanism of MAPI formation, we conducted a set of additional experiments (see Figure 4). First, we investigated how the lead acetate film storage conditions affected the conversion to MAPI. As evident from the two films on the right-hand side in Figure 4 a, storage under ambient lab conditions resulted in an incomplete conversion to MAPI. Meanwhile, the conversion was more complete for the samples stored under dry conditions (left). One explanation for the observed differences could be related to differences in the solubility of the lead acetate layer in the i-PrOH solution. Thus, we also investigated how much of the lead acetate layers dissolved in pure i-PrOH solvent. As can be seen from Figure 4b), the lead acetate film stored under dry conditions dissolves almost completely in i-PrOH. The darker areas are the bare glass substrate (confirmed by EDS, see Figure S4 in the Supporting Information), while the brighter areas consist of a thin (<10 nm) layer of lead species. On the other hand, the sample stored under ambient conditions (Figure 4c) shows a complete coverage with micrometer-sized star-like crystals formed on top. XRR measurements confirm that almost the entire film remains (∼82 nm) after the solubility test.

**Figure 5:**
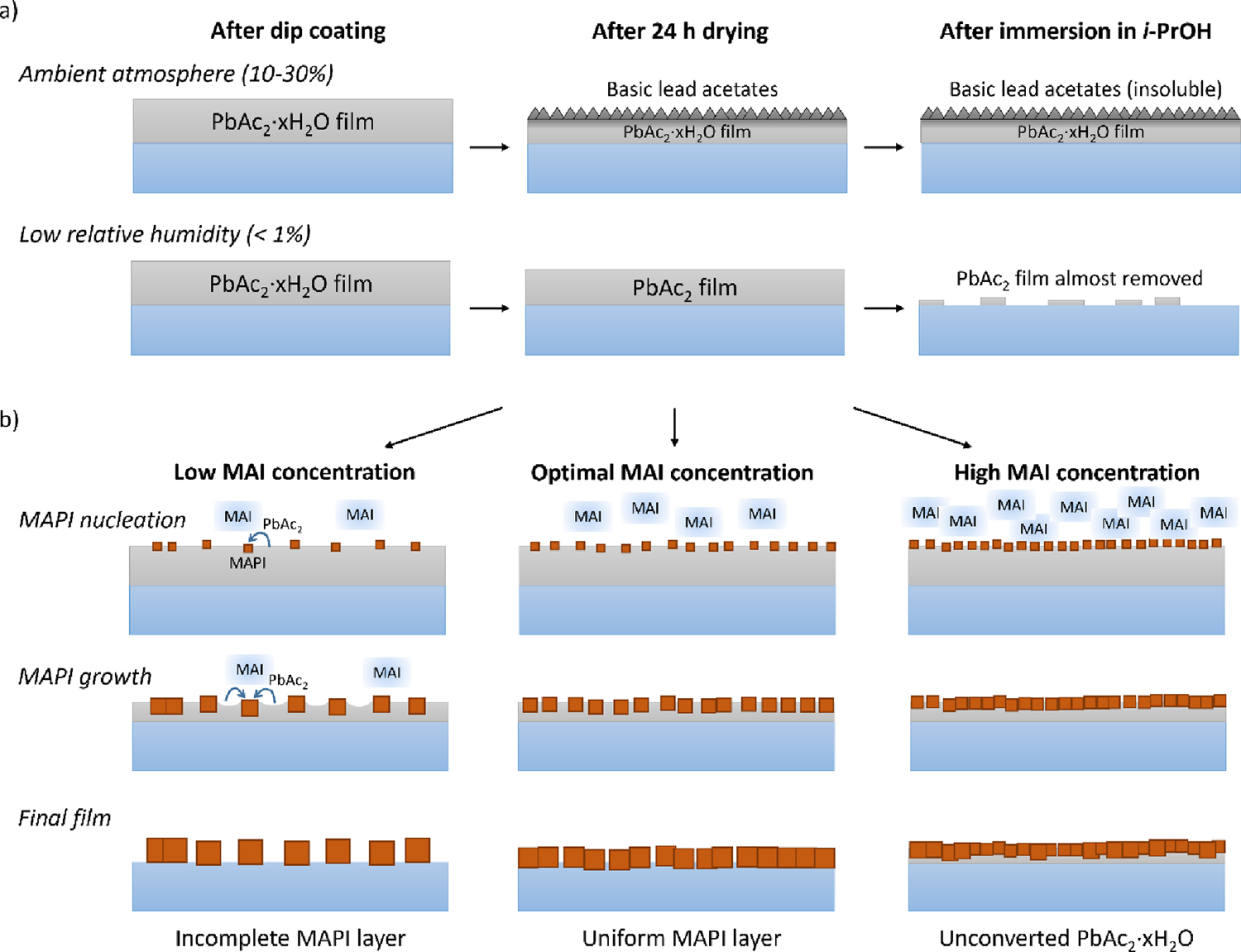
Proposed reaction mechanisms: (a) the effect of humidity on the lead acetate films and (b) the formation mechanism of MAPI.

Thus, the solubility test confirms the formation of an insoluble basic lead acetate layer (possibly 3PbAc_2_·PbO·H_2_O) when the samples are stored in an ambient (slightly humid) atmosphere, which is unfavorable to the formation of MAPI in the next step. However, this can be circumvented by keeping the humidity low (typically below 1%) during the aging of the lead acetate film, which is possible even without glovebox processing. This is schematically illustrated in Figure 5a.

The solubility of the lead acetate layer in i-PrOH is essential to the nucleation and growth mechanism of the subsequent MAPI formation step, as illustrated in Figure 5b. Upon dissolution, the lead acetate molecules rapidly react with MAI molecules in the converter solution. Above a certain threshold (saturation) concentration, heterogeneous nucleation of MAPI will occur on top of the lead acetate film. The number of nucleation sites (and the distance between the nuclei) is directly proportional to the MAI concentration, which is also observed in the SEM images in Figure 2. Subsequent reactions between lead acetate and MAI molecules will not produce new nuclei but will add to the growth of the existing nuclei. The growth process will continue as long as there are available lead acetate species in the solution. In the case of low or intermediate MAI concentrations, the conversion of lead acetate to MAPI will be complete. However, if the MAI concentration is too high (as in the case of 12 mg/mL), part of the lead acetate will be completely blocked by the formed MAPI (which is insoluble in i-PrOH) leading to an incomplete conversion. Furthermore, fewer nuclei formed at low MAI concentrations allows for the growth of larger MAPI grains, as more lead acetate species contribute to the growth process. This is in line with the evolution of the grain size observed in the SEM images in Figure 2. Similar reaction mechanisms have also been described in earlier studies with PbI_2_ used as the lead precursor.^28,39,46,47^ Although we assume that the lead acetate to MAPI conversion is complete at low MAI concentrations, unreacted lead acetate might also be trapped under large MAPI crystals.

### 3.4 Device Performance

**Figure 6:**
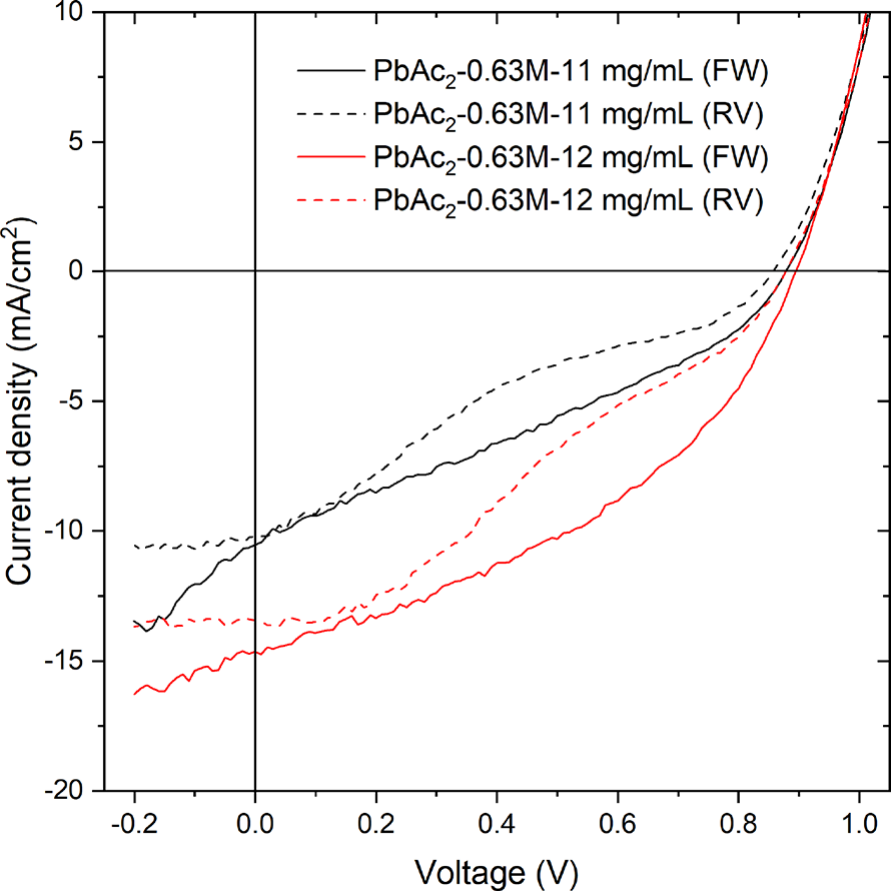
Representative *J*–*V* curves for devices prepared with PbAc_2_-0.63 M and converted to MAPI in 11 or 12 mg/mL MAI solutions (solid lines: forward (FW) bias; dashed lines: reverse (RV) bias).

Finally, the MAPI layers produced via the investigated dip coating method were evaluated in solar cell devices. All the initial processing steps (TiO_2_ deposition, PbAc_2_ deposition, and aging, as well as MAPI conversion) were carried out in dry lab conditions. However, to minimize PbI_2_ formation during the MAPI annealing step, the samples were annealed at 100 °C inside a nitrogen-filled glovebox. For an optimal PSC performance, it is expected that a uniform MAPI layer is needed (see Figure 5b). From the SEM images in Figure 2, it is evident that the formed MAPI structure does not entirely cover the substrate when low MAI concentrations have been used. Thus, we chose to only use the highest MAI concentrations when preparing the devices (i.e., 11 and 12 mg/mL). Representative *J*–*V* curves for these samples are shown in Figure 6, and the extracted device parameters are summarized in Table 2.

**Table 2:** Extracted Device Parameters Are Based on Two Devices per Sample

***forward bias***				
**sample**	*J*_**SC**_(mA/cm^2^)	*V*_**OC**_**(V)**	**FF**	**PCE (%)**
PbAc_2_-0.63M-11 mg/mL	9.28 ± 1.34	0.88 ± 0.02	0.27 ± 0.08	2.66 ± 0.29
PbAc_2_-0.63M-12 mg/mL^a^	14.07 ± 0.81	0.89 ± 0.02	0.41 ± 0.00	5.09 ± 0.38

***reverse bias***				
**sample**	*J*_**SC**_(mA/cm^2^)	*V*_**OC**_**(V)**	**FF**	**PCE (%)**
PbAc_2_-0.63M-11 mg/mL	8.87 ± 2.37	0.86 ± 0.01	0.30 ± 0.02	1.80 ± 0.08
PbAc_2_-0.63M-12 mg/mL	13.68 ± 0.32	0.90 ± 0.02	0.29 ± 0.02	3.51 ± 0.13

a Champion device: PbAc_2_-0.63M-12 mg/mL: *J*_SC_ 14.6 mA/cm^2^, *V*_OC_ 0.90 V, FF 0.41, and PCE 5.36% (in forward bias).

It is evident that the overall device performance improves when the MAI concentration is increased from 11 to 12 mg/mL in the converter solution, as the PCE almost doubles from 2.7 to 5.1% in the forward bias. The main contribution to this comes from an increase in the short-circuit current (*J*_SC_) from 9.3 to 14.1 mA/cm^2^, while the open-circuit voltage (*V*_OC_) is relatively similar ∼0.88–0.89 V. The improved *J*_SC_ can mainly be ascribed to the overall increase in absorbance (especially below 550 nm) as seen in Figure 3. Both samples show an s-shape in the reverse bias, which could be related to problems with the contacts (TiO_2_ or Spiro-OMeTAD),^49^ or to ion migration due to light or applied voltage.^50^

It is evident that these devices do not perform as well as state-of-the-art PSCs. However, we want to stress that the main goal of this study was to show that it is possible to prepare well-defined MAPI films via a scalable dip coating method without the need for glovebox processing and toxic solvents like DMF, as well as to better understand the formation mechanism behind it. Thus, to improve the device performance, further optimization of the method is needed (including finding the optimal lead acetate layer thickness, as well as MAI concentration in the conversion step). The MAPI coverage of the investigated devices was not complete, and a slight increase in the MAI concentration could eliminate any pinholes in the MAPI structure. On the other hand, a too-high MAI concentration would lead to unreacted lead acetate, which could potentially cause the degradation of the MAPI layer into PbI_2_. A hint of PbI_2_ was observed in the UV–vis spectrum for the sample converted in 12 mg/mL MAI (see Figure 3). Furthermore, the formed MAPI grains and crystals are relatively small (∼400 nm) when high MAI concentrations are used. An increase in the crystal size via solvent annealing should also lead to better performance due to fewer grain boundaries.^51^ Finally, more selective contact materials should also lead to better overall device performance. In our case, no mesoporous TiO_2_ layer was utilized on top of the compact TiO_2_, although the porous structure is expected to better encompass the MAPI crystals and, in that way, boost the device performance.^52^ Furthermore, oxygen doping of Spiro-OMeTAD would also lead to a slight improvement in the efficiencies.^53^

## 4 Conclusions

In this study, we investigated the film formation mechanism of MAPI using PbAc_2_·3H_2_O as the precursor. We confirmed that aging the lead acetate film in dry air flow resulted in an anhydrous PbAc_2_ phase that had superior solubility in i-PrOH and resulted in a more complete conversion to MAPI (compared to aging the films under ambient conditions). Furthermore, SEM characterization showed a clear improvement in surface coverage and a decrease in MAPI grain size with increasing MAI concentration. However, at the highest MAI concentration used in this study (12 mg/mL), part of the PbAc_2_ phase was blocked by the formed MAPI layer leading to incomplete conversion and likely to degradation to PbI_2_. Finally, it was shown that working solar cells can be produced with these MAPI films with a clear improvement in power conversion efficiency when the MAI concentration is increased. Further improvements in surface coverage (e.g., via solvent annealing), together with other upgrades on the device fabrication, will boost the performance of the device to make it a realistic candidate for scalable thin film solar cell technology. As the processing steps can be performed under near ambient conditions, the described method will substantially simplify the device fabrication and ultimately lower the production costs.

## Supporting Information Available

XRR data of lead acetate films, topography AFM images and roughness parameters of lead acetate films, particle grain size and surface coverage of MAPI films, higher magnification SEM images of MAPI film, and EDS maps of dissolved lead acetate films (PDF)
