# Supplementary material for: Scalable Lead Acetate-Based Perovskite Thin Films Prepared via Controlled Nucleation and Growth under Near Ambient Conditions
Source: ACS Omega. 2024 Feb 8;9(7):8266–73. doi: 10.1021/acsomega.3c08912 (PMC10882608; doi:10.1021/acsomega.3c08912)
Supplement: Supplementary file 1 — ao3c08912_si_001.pdf [file ao3c08912_si_001.pdf]

Scalable lead acetate-based perovskite thin films prepared via controlled nucleation and growth under near ambient conditions

Supporting information

*Saara Sirkiä,[a] Muhammad Talha Masood,[b] Mahboubeh Hadadian,[c] Syeda Qudsia,[a] Emil Rosqvist,[a] Jan-Henrik Smått [a,]**

[a] Laboratory of Molecular Science and Engineering, Åbo Akademi University, Henriksgatan 2, FI-20500 Åbo, Finland.

[b] Department of Materials Engineering, School of Chemical & Materials Engineering, National University of Science & Technology (NUST), H 12 sector, 44000, Islamabad, Pakistan.

[c] Department of Mechanical and Materials Engineering, Faculty of Technology, University of Turku, FI-20014 Turku, Finland.

* Corresponding author, contact: jan-henrik.smatt@abo.fi

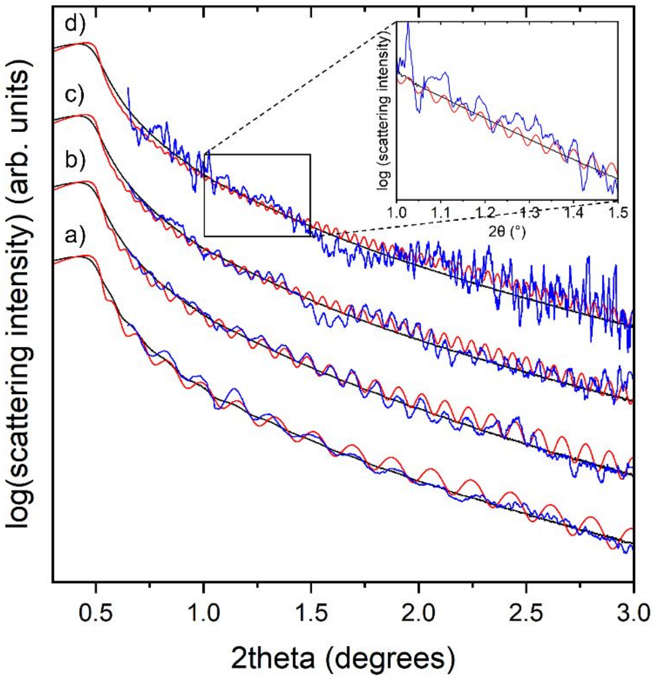

Figure S1. XRR data of the produced lead acetate films: a) PbAc2-0.24M, b) PbAc2-0.44M, c) PbAc2-0.63M, and d) PbAc2-0.81M. This includes the original XRR data (in black), the amplified and smoothed data (in blue), as well as the simulated data (in red). The inset shows a higher magnification of the PbAc2-0.81M sample.

As evident from the original data plots, the Kiessing fringes are very difficult to observe and analyze (black curves). This is probably due to the small density difference between the lead acetate film and the glass substrate. To make it easier to analyze, we first amplified the signal and then smoothened the resulting data (Savitzky-Golay method) using the Origin 2022

software (see curves in blue). The amplification and smoothing of sample PbAc2-0.24M were 10 times and 15 pt, respectively. 30 times and 25 pt were used for sample PbAc2-0.44M, 50 times and 25 pt were used for sample PbAc2-0.63M, and 100 times and 15 pt were used for sample PbAc2-0.81M.

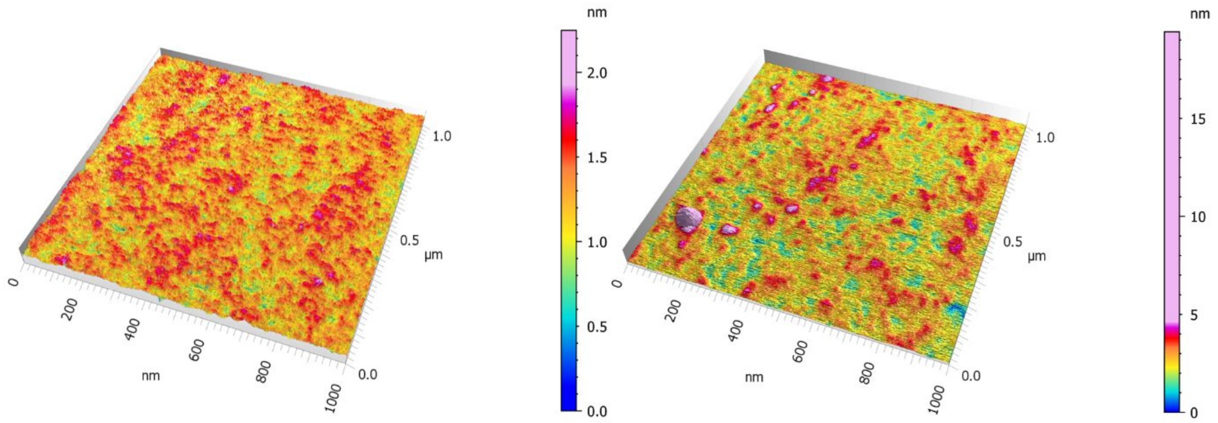

Figure S2. *Topography AFM (1 µm x 1 µm) images of $PbAc_2$-0.63M samples aged under a) dry conditions for 24 h, and b) ambient conditions for 24 h.*

To thoroughly report the surface character of the surfaces, the seven roughness parameters were analyzed (Table S1) [1]. First are the amplitude parameters S_a and S_q, which give the average deviation from the mean level of the surface as the arithmetic, respectively the root-mean-square average. The distribution of heights is described by the skewness (S_{sk}) and kurtosis (S_{ku}). The skewness gives the asymmetry of distribution, a positive S_{sk} describing a peak dominated surface (as was typically observed for these surfaces). Kurtosis describes the broadness of height distribution, a value of 3 being mesokurtic (the case of the dry store sample at 1 µm x 1 µm), the distribution of heights being following a normal distribution. Higher S_{ku} values indicate a higher peak and taller tails. The S_{al} is the autocorrelation length and could be seen as a measure of lateral roughness (compared to amplitude roughness). A lower value indicates more closely spaced features and vice versa. The parameter S_{dr} is the developed surface area ratio which describes the percentual increase in surface area due to roughness, in comparison to a perfectly flat surface. Finally, S_{pd} gives the point density, i.e., peaks per surface area, discriminating peaks surrounded by lower data points after a segmentation (Wolf pruning at 5% of the ten-point height).

Table S1. *Roughness parameters determined from 10 µm x 10 µm and 1 µm x 1 µm AFM images, with standard deviations [1].*

	Sample stored dry (RH <1%)				***Sample stored in ambient***			
**Parameter**	**1 µm**	**StDev**	**10 µm**	**StDev**	**1µm**	**StDev**	**10 µm**	**StDev**
**S_a(nm)**	0.15	±0.01	0.21	±0.03	0.58	±0.17	1.02	±0.38
**S_q (nm)**	0.19	±0.02	0.32	±0.10	0.90	±0.29	1.91	±1.06
**S_{sk} (-)**	0.00	±0.05	2.32	±3.19	2.29	±1.25	3.10	±2.44
**S_{ku} (-)**	3.13	±0.06	30.1	±38.2	24.5	±23.6	27.6	±18.8
**S_{al} (nm)**	25.1	±2.7	365	±136	43.1	±26.1	483	±309
**S_{dr} (%)**	0.39	±0.11	0.01	±0.001	1.45	±0.73	0.17	±0.06
**Spd (1/µm^2)**	46.8	±19.1	58.8	±37.9	5.4	±5.1	15.0	±8.9

Table S2. *Properties of MAPI film prepared from converting $PbAc_2$-0.63M in different MAI concentrations.*

MAI concentration (mg/mL)	Particle grain size (µm)	Surface coverage (%)
9.0	0.84 ± 0.06	72.0
9.5	0.68 ± 0.07	74.5
10.0	0.64 ± 0.05	80.2
10.5	0.55 ± 0.06	82.6
11.0	0.47 ± 0.04	89.2
12.0	0.41 ± 0.05	91.8

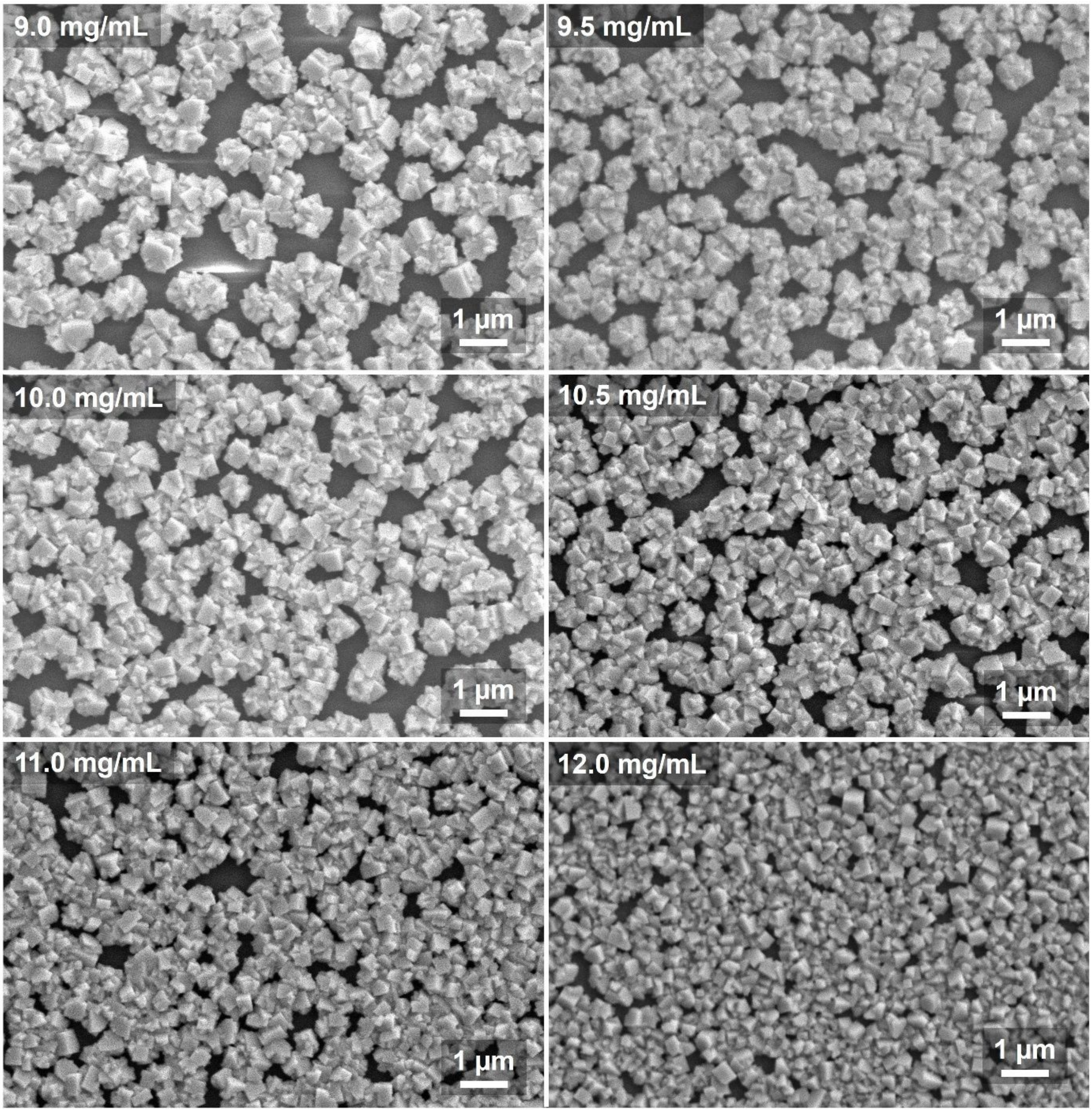

Figure S3. *SEM images with higher magnifications of $PbAc_2$-0.63M samples converted to MAPI in MAI solutions of various concentrations.*

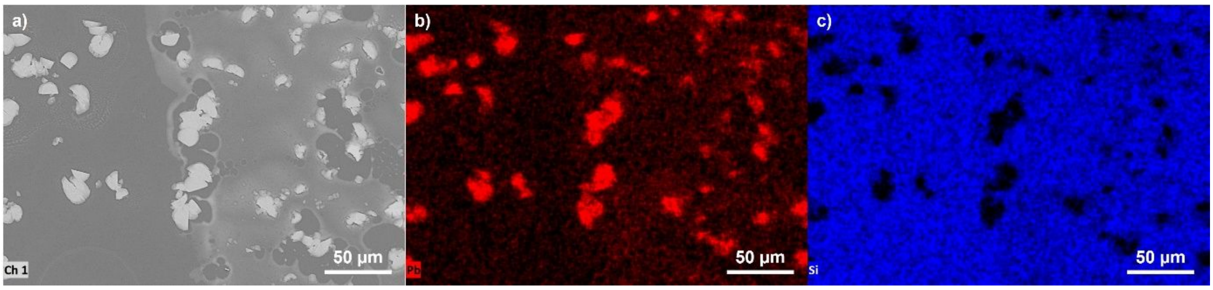

Figure S4. *SEM image (a) and the corresponding Pb (b) and Si (c) EDS maps of lead acetate films after aging under dry atmosphere for 24 h and then immersing them in i-PrOH.*

References

[1] D. J. Whitehouse, *Handbook of Surface and Nanometrology*, 2nd ed. Boca Raton: CRC Press, 2010.
